# Quantitative Proteomics Identify Novel miR-155 Target Proteins

**DOI:** 10.1371/journal.pone.0022146

**Published:** 2011-07-25

**Authors:** Christopher Lößner, Jan Meier, Uwe Warnken, Michael A. Rogers, Peter Lichter, Armin Pscherer, Martina Schnölzer

**Affiliations:** 1 Functional Proteome Analysis, German Cancer Research Center (DKFZ), Heidelberg, Germany; 2 Division of Molecular Genetics, German Cancer Research Center (DKFZ), Heidelberg, Germany; The Research Institute for Children, United States of America

## Abstract

**Background:**

MicroRNAs are 22 nucleotides long non-coding RNAs and exert their function either by transcriptional or translational inhibition. Although many microRNA profiles in different tissues and disease states have already been discovered, only little is known about their target proteins. The microRNA miR-155 is deregulated in many diseases, including cancer, where it might function as an oncoMir.

**Methodology/Principal Findings:**

We employed a proteomics technique called “stable isotope labelling by amino acids in cell culture” (SILAC) allowing relative quantification to reliably identify target proteins of miR-155. Using SILAC, we identified 46 putative miR-155 target proteins, some of which were previously reported. With luciferase reporter assays, CKAP5 was confirmed as a new target of miR-155. Functional annotation of miR-155 target proteins pointed to a role in cell cycle regulation.

**Conclusions/Significance:**

To the best of our knowledge we have investigated for the first time miR-155 target proteins in the HEK293T cell line in large scale. In addition, by comparing our results to previously identified miR-155 target proteins in other cell lines, we provided further evidence for the cell line specificity of microRNAs.

## Introduction

MicroRNAs (miRNAs, miRs) are endogenous, non-coding, single-stranded RNA molecules of about 22 nucleotides that regulate gene expression at the post-transcriptional level [1]. A total of 721 different miRNAs are known so far in humans (miRBase, release 14), and it is assumed that they influence around 60% of protein-coding genes [2], affecting almost every cellular process [3]. Mature miRNAs are incorporated into the RNA Induced Silencing Complex (RISC), which guides them to the appropriate mRNAs [4]. This interaction results in either the degradation of the mRNA or the inhibition of protein translation [5]–[16]. In the latter case, possible points of action are the initiation [8], [9], [10] or elongation step [11] of protein translation. The changes at the protein level are often subtle, leading to the proposed function of microRNAs as rheostats [17].

High proteome coverage and sensitivity are necessary for large scale microRNA target protein detection. A method called “stable isotope labelling by amino acids in cell culture” (SILAC) has emerged as a powerful approach for quantitative proteomics in the field of microRNA target identification in cell culture models [5], [7], [16], [18]. In the SILAC approach, different cell populations are metabolically labelled to saturation with either light or heavy isotopic amino acids. The isotopically labelled proteins are subsequently resolved, identified and quantified by mass spectrometric analysis [19].

For a few target proteins, cell line specificity of microRNA action has been shown [20]. Unfortunately, due to the fact that the majority of large scale quantitative proteomic data for miRNA target protein detection were performed in HeLa cells [5], [7], [18], few conclusions can be drawn regarding target proteins in other cell lines.

In this paper, we focus on human microRNA 155 (hsa-miR-155), which is known to influence many diseases and is assumed to function as an oncoMir in cancer [21]. MiR-155 is derived from a non-coding RNA transcribed from the B-cell Integration Cluster (BIC) located on chromosome 21 [22]. In contrast to the considerable knowledge concerning the deregulation of miR-155, only a few target proteins have been validated for miR-155 so far [21].

In order to identify miR-155 targets with large scale proteomic techniques, we analysed HEK293T cells overexpressing miR-155 using the SILAC method. To address the issue of cell line specificity, we compared our data with recently published SILAC data of miR-155 targets in HeLa cells [5]. In addition, we bioinformatically examined the influence of the identified target proteins on biological processes using functional annotation enrichment analysis.

## Materials and Methods

### Cloning

In order to over express miR-155, we cloned a 165 bp fragment of the BIC gene containing the miR-155 sequence into the EcoRI-XhoI site of the pCMX-PL1 vector (pCMX-miR-155), using the primer pair found in Table S1. The pCMX-PL1 vector was a generous gift of Dr. Roland Schüle (Women's Hospital and Center for Clinical Research, Medical School Freiburg, Germany).

Luciferase sensor constructs were generated by cloning full length 3′UTRs containing the predicted miR-155 binding site into a pMIR-REPORT vector (Ambion, Lincoln, USA) using the primers found in Table S1.

### Cell culture and transfection

For SILAC analysis, SILAC D-MEM media supplemented with 10% dialyzed FBS and either 100 mg/L ^12^C_6_-L-arginine and ^12^C_6_- L-lysine or ^13^C_6_-L-arginine and ^13^C_6_-L-lysine (Invitrogen Corporation, Carlsbad, USA) as well as 200 mg/L ^12^C_5_-L-proline (Promega Corporation, Madison, USA) were used [23]. HEK293T cells were serially passaged (2×10^6^ cells/10 cm dish) and grown for five doublings to ensure full incorporation of labelled amino acids. Subsequently, the cells were transfected with either 2 µg of the pCMX-miR-155 construct or the empty plasmid. The cells were harvested 48 h after transfection and counted using a cell counter (Vi-CELL XR; Beckman Coulter, Fullerton, USA). Aliquots of cells were mixed in a one to one ratio, washed two times with ice-cold phosphate-buffered saline (PBS), shock-frozen in liquid nitrogen for storage at −80°C. A technical and biological replicate of the same experiment was done with reverse labelled samples.

For all other transfection assays, HEK293T cells (5×10^4^/24 well plate) were cultured at 37°C and 10% CO_2_ in D-MEM medium supplemented with 100 units/mL penicillin, 100 µg/mL streptomycin (Invitrogen, Carlsbad, USA) and 10% fetal bovine serum (FBS) (Biochrom, Berlin, Germany). Cells were transfected with the respective vector plasmid (see below) using TransIT LT1 transfection reagent (Mirus Bio LLC, Madison, USA) according to the manufacturer's instructions. Cells were harvested 48 h after transfection.

### Small RNA preparation and control of miR-155 expression

For verification of miR-155 overexpression, small RNAs were extracted using a miReasy Kit (Qiagen, Hilden, Germany). Quantification of miRNA expression was performed using the TaqMan microRNA Assay Kit (Applied Biosystems, Foster City, USA) following the manufacturer's instructions. Each sample was analysed in triplicate using an ABI PRISM 7700 quantitative PCR apparatus (Applied Biosystems, Foster City, USA). The expression values were normalized against the small housekeeping RNAs RNU-6B and RNU-66. A standardized reference of total RNA (Stratagene, La Jolla, USA) was used for standard curve normalization.

### Cell lysis and determination of protein concentration

Frozen cell pellets were lysed using RIPA buffer (50 mM Tris-HCl pH 7.5; 150 mM NaCl; 1% Triton X-100; 0.5% Na-deoxycholate; 0.1% SDS; 10 mM N-ethylmaleimide, supplemented with mini complete EDTA-free protease inhibitor cocktail tablet (Roche Diagnostics, Mannheim, Germany) [24]. Protein concentration of supernatants was measured by 2D Quant Kit (GE Healthcare Bio-Sciences, Uppsala, Sweden).

### SDS-PAGE, tryptic digestion and mass spectrometric analysis

Equal amounts of protein (100 µg) of each forward and reverse labelled sample were precipitated using the chloroform/methanol method [25] and separated on a 4–12% SDS polyacrylamide gel (NuPAGE Novex Bis-Tris Gel; Invitrogen Corporation, Carlsbad, USA) following the manufacturer's instructions. The entire lanes were cut to get 26 gel slices. Tryptic digestion and extraction were performed as previously described [26] with adaption to the volume of the gel plugs. After extraction, the samples were vacuum dried and then dissolved in 0.1% TFA.

Half of the sample was used for nanoHPLC-ESI-MS/MS analysis using a LC Packings Ultimate (Dionex Corporation, Sunnyvale, USA) and a LTQ Orbitrap XL (Thermo Fisher Scientific, Waltham, USA). A Reprosil-PUR C18-AQ, 3 µm, 1500×0.075 mm column (Dr. Maisch HPLC GmbH, Ammerbuch-Entringen, Germany) and a gradient ranging from 14 to 86% MeCN (in 0.1% FA and HPLC-H_2_O) over 90 min was used for nanoHPLC. Electrospray ionisation was achieved by using a PicoTip Emitter Silica Tip (New Objective, Woburn, USA) and a spray voltage of 1.7 kV. Data dependent acquisition using Xcalibur 2.0.6 (Thermo Fisher Scientific, Waltham, USA) was performed by one FTMS scan with a resolution of 60000 (m/z 400) and a range from 370 to 2000 m/z and 6 MS/MS scans of the most intense precursor ions in the ion trap.

### Protein identification and quantification of MS data

For identification and quantification of peptides and their respective proteins, MaxQuant 1.0.13.13 [27] was used with the human IPI database (version 3.65; www.ebi.ac.uk/IPI) and Mascot 2.2.04 (Matrix Science, London, UK). Standard settings were used for MaxQuant [28], except that the filtering of labelled amino acids was prohibited and protein groups with at least one ratio count (evidences of different peptides, charge states or measurements in replicate) were considered. As identified, all protein groups with a false discovery rate below 1%, without contaminants and decoy entries as well as groups without “only identified by site” were chosen. Quantified protein groups were all identified ones with at least three ratio counts. Finally, as regulated protein groups, the quantified ones with a “significance B” value (indicating whether a ratio is significantly different from the distribution of all protein ratios; binned into groups by intensity) of less than 0.01 and a variability of less than 50% were considered. Information on all quantified proteins which are down- and upregulated is listed in Table S2 and S3.

### Western blot analysis

For Western blotting, protein extracts of non-SILAC labelled HEK293T cells transfected with 0.45 µg pCMX-miR-155 or the empty vector were prepared, 10 or 20 µg of protein lysates were precipitated and separated by SDS-PAGE as described above.

Tank blotting was performed using the XCell II Blot Module (Invitrogen Corporation, Carlsbad, USA) according to the manufacturer's instructions. For immune staining, ECL Plex (GE Healthcare Bio-Sciences, Uppsala, Sweden) was employed using CyDye coupled secondary antibodies in conjunction with the primary antibodies as stated in Table S1. Blots were scanned using a Typhoon 9410 scanner (GE Healthcare Bio-Sciences, Uppsala, Sweden) and the instructions described in the ECL Plex manual. For quantification of the resulting images, ImageJ version 1.4 (http://rsb.info.nih.gov/ij/) was used. The intensity values of the target protein signals were normalized to the intensity value of GAPDH as a housekeeping protein.

### qRT-PCR of potential miR-155 targets

HEK293T cell were transiently transfected with either 0.45 µg of the pCMX-miR-155 construct or the empty vector. MicroRNA containing total RNA was prepared using the miReasy RNA isolation kit (Qiagen, Hilden, Germany). DNase 1 predigestion and random primed first strand synthesis was performed on 2 µg of the total RNA using a slight modification of the SuperscriptII First Strand Synthesis protocol (300 ng random hexamers, RNaseH treatment eliminated, 2 µg of T4 gene 32 protein (single stranded DNA binding protein added; Invitrogen Cooperation, Carlsbad, USA). Two microliter of each cDNA sample was analysed in triplicate with the ABI PRISM 7900HT (Applied Biosystems Inc, Foster City, USA) using Absolute SYBR Green ROX Mix (ABgene, Hamburg Germany) according to the manufacturer's instructions. Primers for qRT-PCR analysis were designed using the Universal ProbeLibrary Assay Design Center (www.roche-applied-science.com), and possible transcript variants were considered. The primers used for analysis, as well as the two endogenous housekeeping genes (PGK1, DCTN2) used as internal standards, are found in Table S1. Cycling conditions for the assay were 50°C, 2 min; 95°C, 15 min; thereafter 40 cycles of 95°C, 15 sec, 60°C, 1 min; thereafter 95°C, 15 sec; 60°C, 15 sec; 95°C, 15 sec. The relative quantification of the RNA of interest in comparison with the housekeeping genes was calculated by using standard curves for each gene.

### Luciferase reporter assay

The pMIR-REPORT luciferase sensor constructs (5 ng) were co-transfected with a TK *renilla* expression plasmid (45 ng, Promega, Madison, USA) and pCMX-miR-155 (450 ng) into HEK293T cells using TransIT LT1 transfection reagent as described above (Mirus Bio LLC, Madison, USA). As a negative control, HEK293T cells were co-transfected with an empty pCMX vector (450 ng) instead of the pCMX-miR-155. The cells were harvested and lysed at 48 h post-transfection. Luciferase signals were measured using a standard dual luciferase buffer system [29] on a Mithras luminescence reader (Berthold Technologies, Bad Wildbad, Germany). Each sample was measured in triplicate and six assays per sample were performed.

### Mapping of prediction algorithm data

Target genes for miR-155 were predicted using TargetScan v5.1 [2], [30], [31], RNA22 5′UTR, 3′UTR and CDS [32], MicroCosm Version 5 [33] and DIANA-microT v3.0 [34]. The 5 top IPI IDs of the SILAC quantified proteins were mapped by BRM [35] using their Ensembl gene IDs, Ensembl transcript IDs or HGNC gene symbols (ENSEMBL 56 Genes (Sanger UK) Homo sapiens genes (GRCh37)) using the cross references obtained by the web interface of BioMart [36].

### Identification of seed sequence matches

The 8mer (UUAAUGCU), 7mer-A1 (UUAAUGC) and 7mer-m8 (UAAUGCU) seed sequences of miR-155 were used to search (FuzzNuc, www.emboss.org) for complete complementary in the 5′UTR, CDS and 3′UTR of the proteins identified as upregulated, unregulated and downregulated in SILAC quantification. Thereafter, the TOP IPI identifier was used to obtain the corresponding ENST identifiers using the web interface of BioMart [36] (ENSEMBL 56 Genes (Sanger UK) Homo sapiens genes (GRCh37)). The sequences of the 5′UTR, CDS and 3′UTR were acquired from the human Ensembl database (www.ensembl.org) using Ensembl API 52.

### Functional annotation clustering

For functional annotation clustering, the beta version of DAVID 2008 was used [37], [38]. The TOP IPI IDs of the downregulated protein groups were compared against all quantified protein groups of the SILAC experiment as background. The functional annotation clustering was conducted using all gene ontology annotated biological processes at standard settings.

## Results

### Detection of miR-155 regulated proteins by SILAC

In order to detect miR-155 regulated proteins, we ectopically expressed miR-155 in HEK293T cells and compared the effects at the protein level by quantitative proteomics using SILAC.

**Figure 1:**
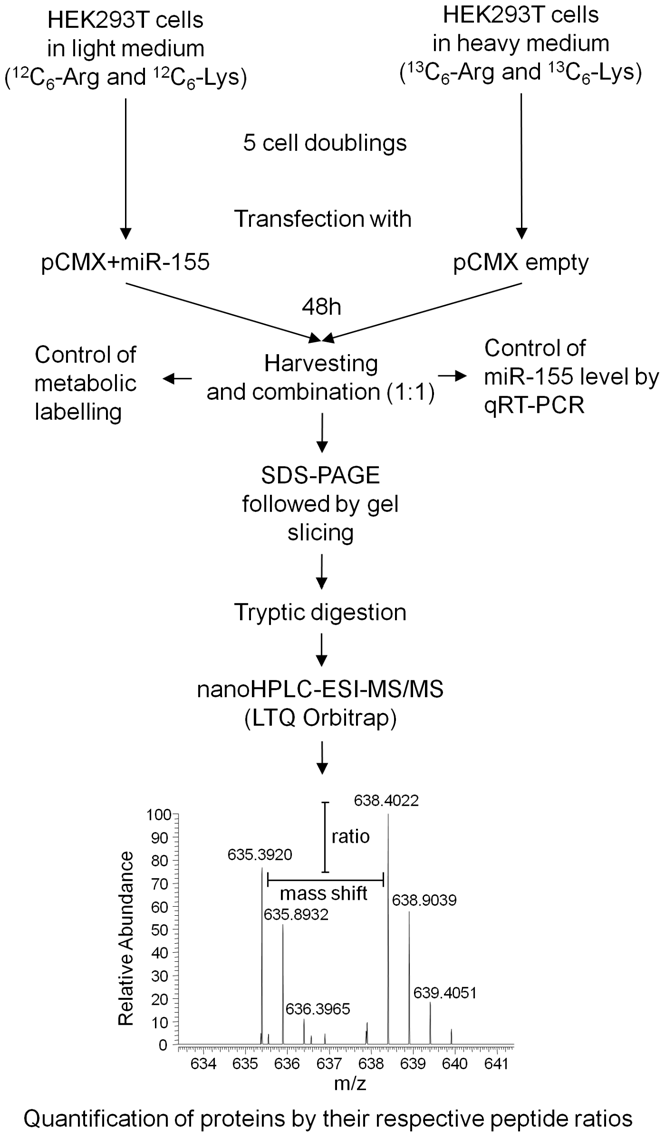
Experimental setup of SILAC quantification.

The level of miR-155 was manipulated by overexpressing miR-155 under the control of a viral promoter using the pCMX vector system, which resulted in a ca. 1000 fold overexpression when compared to the low endogenous level of miR-155 in HEK293T cells containing the empty vector controls as determined by qRT-PCR (see Figure S1). The total proteomic analysis workflow is depicted in Figure 1.

**Figure 2:**
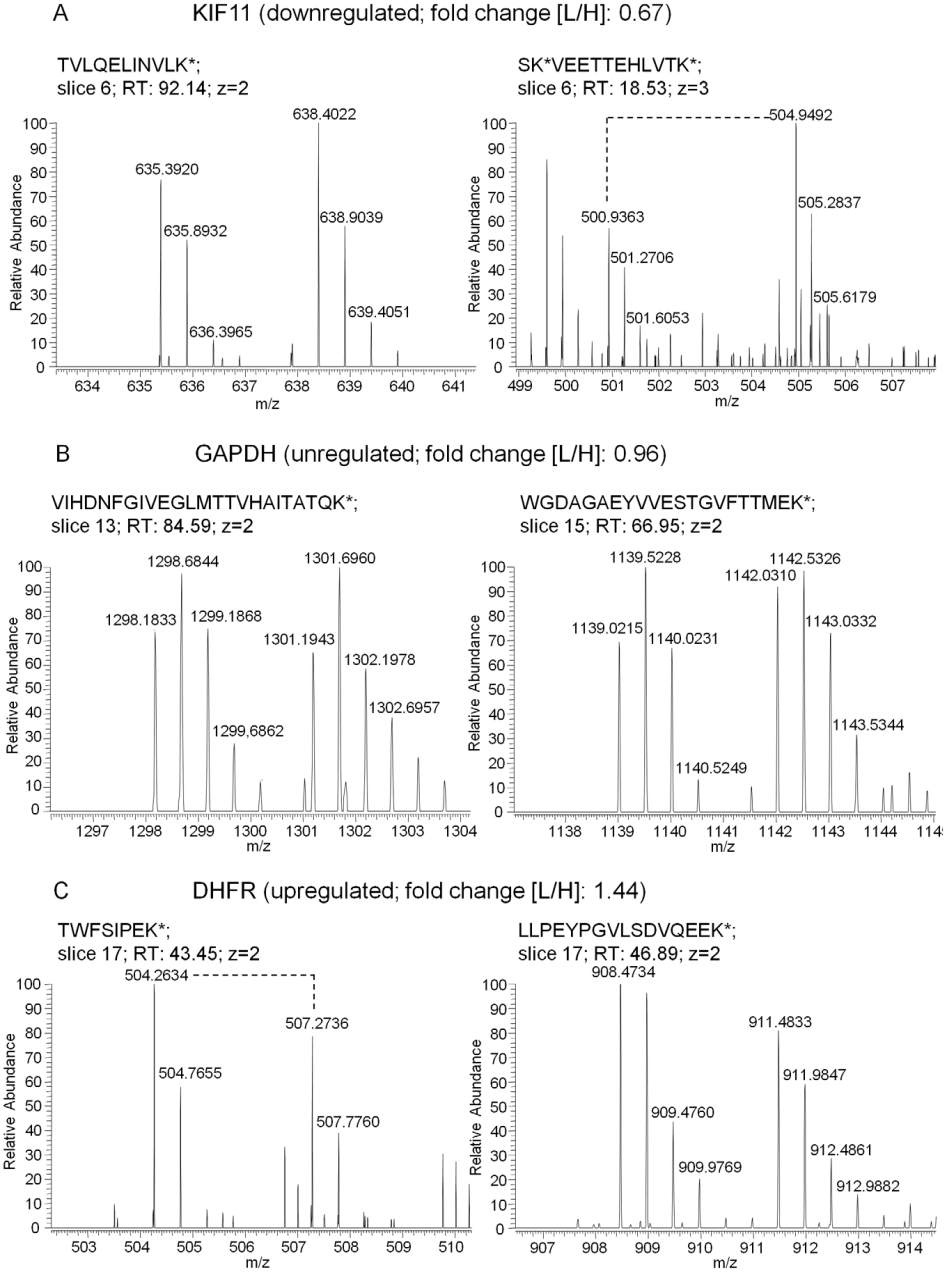
Examples of SILAC quantified peptides. The relative abundance (y-axis) is shown for SILAC peptide pairs and their m/z (mass to charge ratio) values (x-axis). For each peptide the sequence is displayed and labelled amino acids are marked by an asterisk (*). The slice number of the SDS-PAGE gel, retention time (RT) in minutes of the peptide in the C18-RP nanoHPLC run and the charge state (z) are also depicted. All peptides have mass shifts of 6.02 Da per labelled amino acid. **A.** KIF11, as an example for a downregulated protein by miR-155 overexpression, is highlighted by two peptides. Both peptides have a higher relative abundance for the heavy labelled form. **B.** For GAPDH both peptides display a fairly equal relative abundance. **C.** DHFR has a higher relative abundance for the light labelled form due to miR-155 overexpression.

Using the SILAC approach, we could identify a total of 3148 protein groups and quantified 2434. Eighty-eight protein groups were differentially expressed; whereof 42 were upregulated and 46 downregulated (information on down- and upregulated proteins is given in Table S2 and S3). In addition details on five selected proteins and their respective peptides are listed in Table S4). The downregulation of the potential miR-155 target proteins ranged from 0.58 to 0.85 fold (18 to 72% downregulation) with an average value of 0.76 (32%). Figure 2 exemplifies a downregulated protein, KIF11 (A), an unaffected protein, GAPDH (B) and an upregulated protein, DHFR (C). These results reveal a set of novel miR-155 regulated target proteins in HEK293T cells.

### Western blot analysis of downregulated proteins

**Figure 3:**
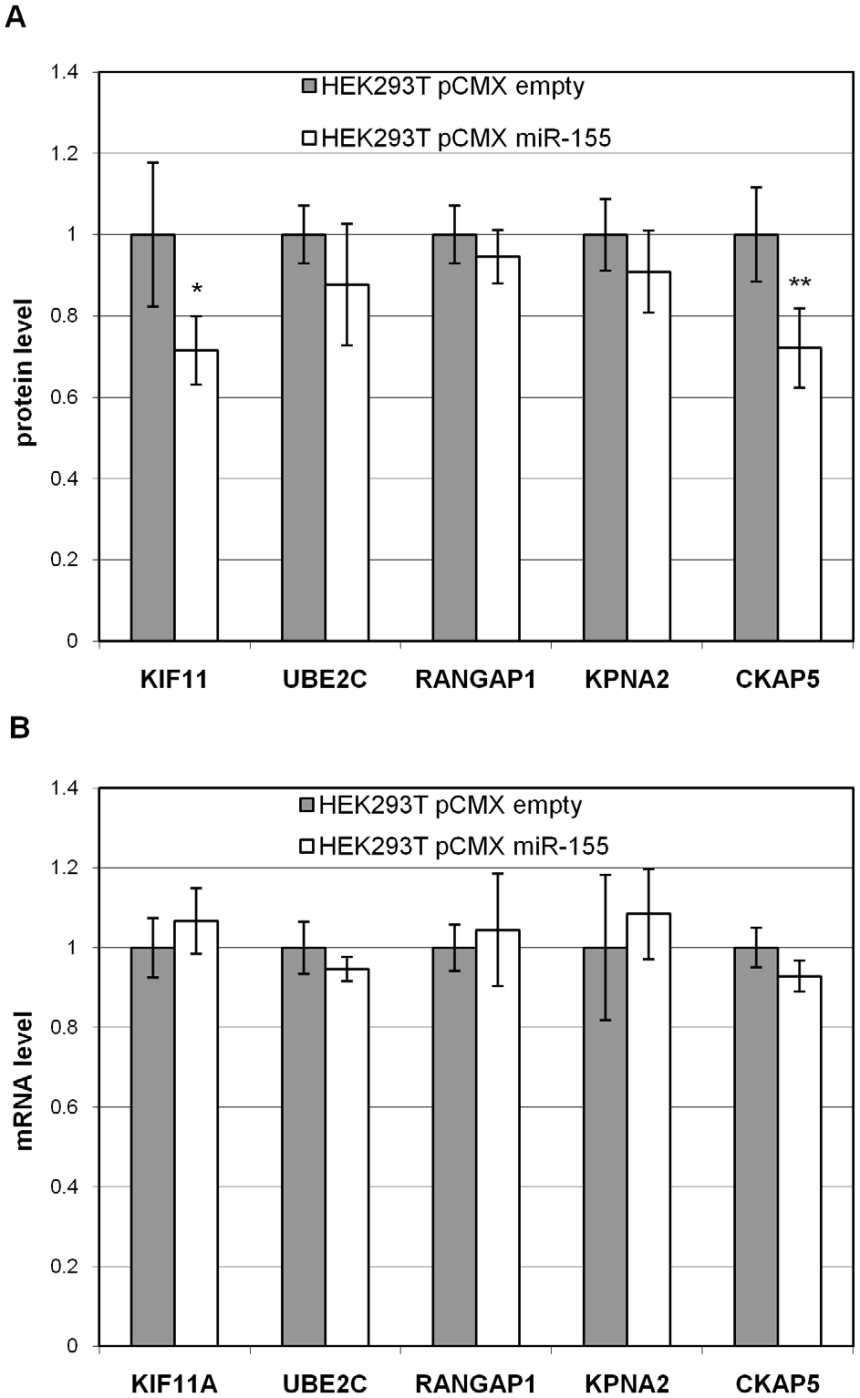
Western blot analysis and qRT-PCR analysis of five selected target proteins. The protein levels **A** or mRNA levels **B** of the miR-155 overexpression (HEK293T pCMX miR-155) are compared to the control (HEK293T pCMX empty). The values of the controls are set to 1. The standard bars show the standard deviation between the four **A** or three **B** biological replicates. *: Student's t-test<0.05; **: Student's t-test<0.01.

To validate the miR-155 targets detected by SILAC at the protein level, we analysed their regulation by quantitative Western blot analysis using the ECL Plex system on four biological replicates, independent of the SILAC analysis. For Western blot analysis, we selected five proteins using 3 criteria: only positive by seed sequence matches (KIF11), positive by prediction methods and seed sequence matches (CKAP5), and neither positive by seed sequence matches nor by prediction methods (UBE2C, RANGAP1, and KPNA2). The results of the five tested targets, KIF11, UBE2C, RANGAP1, KPNA2 and CKAP5 are shown in Figure 3 A. It was confirmed that all five proteins showed a lower expression which was statistically significant for CKAP5 (p<0.01, see also Figure S2) and KIF11 (p<0.05). A high correlation of fold changes obtained by SILAC versus fold changes obtained by Western blot analysis was seen (see Table 1), and allowed a verification of our SILAC quantification.

**Table 1:** Potential downregulated miR-155 target proteins in HEK293T cells detected by SILAC with supportive data (Western blot analysis, qRT-PCR, miRNA binding).

	Protein level		mRNA level	miR-155 binding to mRNA sequences		
HGNC symbol	SILAC	Western blot	qRT-PCR	Luciferase reporter assay	Prediction algorithms	Seed sequence matches
IMP3	0,579	-	-	-	n.d	n.d.
MRFAP1	0,594	-	-	-	n.d.	3′UTR7mer-A1
CENPE	0,623	-	-	-	n.d.	n.d.
PCM1	0,634	-	-	-	n.d.	CDS 7mer-m8
HMGCS1	0,638	-	-	-	RNA22-CDS	3′UTR 8mer
MAN2B1	0,644	-	-	-	n.d.	n.d.
KIF11	0,675	0.7*	1.07	1.16	n.d.	CDS 7mer-A1
TNPO3	0,677	-	-	-	n.d	n.d.
FAM115B	0,699	-	-	-	n.d.	CDS 7mer-A1, CDS 7mer-m8 3′UTR
UBE2C	0,742	0.84	0.95	-	n.d.	n.d.
POP7	0,743	-	-	-	n.d	n.d.
SRXN1	0,744	-	-	-	n.d.	3′UTR 7mer-m8
MAPK14	0,745	-	-	-	n.d.	n.d.
GPS1	0,746	-	-	-	n.d.	n.d.
GEMIN4	0,747	-	-	-	n.d.	3′UTR 7mer-m8
KPNA2	0,748	0.9	1.08	-	n.d.	n.d.
SSR1	0,75	-	-	-	RNA22-3′UTR	3′UTR 7mer-A1
NOB1	0,751	-	-	-	n.d.	n.d.
RBM4	0,754	-	-	-	n.d.	n.d.
COPS3	0,757	-	-	-	n.d.	3′UTR 7mer-A1
TIMM17A	0,764	-	-	-	n.d.	n.d.
GIGYF2	0,769	-	-	-	n.d.	CDS 7mer-A1
BAG6 (BAT3)	0,774	-	-	-	n.d.	n.d.
HSPBP1	0,778	-	-	-	n.d.	n.d.
HSPA1A	0,778	-	-	-	n.d.	n.d.
DNAJA1	0,779	-	-	-	n.d.	n.d.
USP14	0,78	-	-	-	n.d.	3′UTR 7mer-m8
AASDHPPT	0,787	-		-	RNA22-CDS.	n.d.
GTF3C5	0,787	-	-	-	n.d.	n.d.
RANGAP1	0,788	0.91	1.04	-	n.d.	n.d.
METAP1	0,792	-	-	-	n.d.	n.d.
EIF1	0,796	-	-	-	n.d.	n.d.
CSDE1	0,798	-	-	-	n.d.	n.d.
RPL39	0,8	-	-	-	n.d.	n.d.
TUBA1C	0,815	-	-	-	n.d.	CDS 7mer-A1
HEATR1	0,817	-	-	-	n.d.	n.d.
SRRM2	0,817	-	-	-	n.d.	n.d.
ABCF2	0,818	-	-	-	n.d.	n.d.
RBM3	0,821	-	-	-	n.d.	n.d.
CKAP5	0,835	0.74**	0.93	0.65*	RNA22-CDS DIANA microT Targetscan	CDS 8mer, 3′UTR 7mer-A1
DDX5	0,84	-	-	-	n.d.	n.d.
DDX3X	0,843	-	-	-	n.d.	n.d.
PDCD5	0,843	-	-	-	n.d.	n.d.
EIF4A1	0,843	-	-	-	n.d.	n.d.
RPS14	0,844	-	-	-	n.d.	n.d.
RPS8	0,849	-	-	-	n.d.	n.d.

_a_HGNC gene symbols (www.genenames.org);

_b_fold change miR-155/pCMX;

_c_RNA22 CDS, 3′UTR, 5′UTR; DIANA microT, TargetScan, MicroCosm;

_d_Seed sequence (8mer, 7mer-A1, 7mer-m8) matches in the different mRNA regions (5′UTR, CDS, 3′UTR); -: not tested; n.d.: not detected;

*Student's t-test below 0,05;

**Student's t-test below 0,01.

### qRT-PCR analysis of genes of downregulated proteins

To compare miR-155 regulatory effects at the protein level with those at the mRNA level, we conducted qRT-PCR analysis for the genes of the five proteins determined by SILAC and verified by Western blot analysis. No downregulation of the tested genes was seen at the mRNA level (see Figure 3 B). This result indicated that the respective mRNAs are not degraded and miR-155 acts predominantly via inhibition of protein translation.

### Binding of miR-155 to putative targets

**Figure 4:**
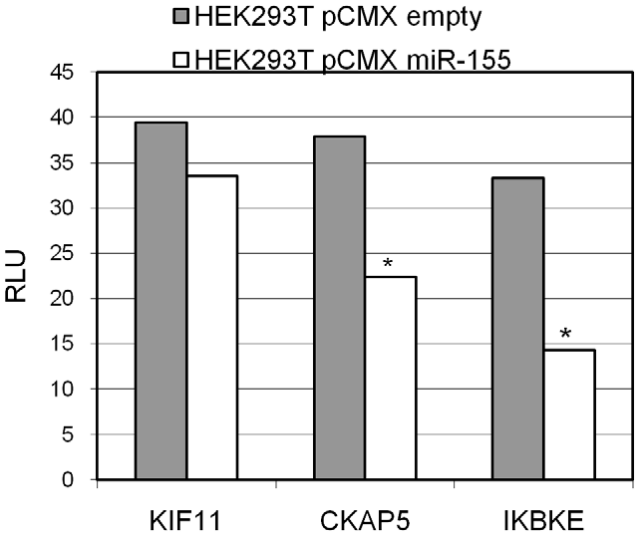
Luciferase reporter assays of the 3′UTR regions of KIF11, CKAP5 and IKBKE. The average relative light units (RLU) of six biological replicates are compared between miR-155 overexpression (HEK293T pCMX miR-155) and control (HEK293T pCMX empty). *: Student's t-test<0.05.

We compared bioinformatically the complete set of 46 potential miR-155 targets using different prediction algorithms (RNA22 CDS, 3′UTR, 5′UTR; DIANA microT, TargetScan and MicroCosm) and analysed the seed sequence matches (8mer, 7mer-A1 and 7mer-m8) of miR-155 in the 5′UTR, CDS and 3′UTR sequences of our targets. The detection of CKAP5 by the prediction algorithm DIANA microT, TargetScan, RNA22 and the seed sequence matches 7mer-A1 in the 3′UTR supported the luciferase reporter assay result (see Figure 4). Beside the binding of miR-155 to the 3′UTR of CKAP5, there was also a putative binding site in the CDS region indicated by RNA22-CDS and one 8mer seed sequence match (Table 1). For KIF11 there was no indication for 3′UTR binding by prediction algorithms or seed sequence matches, and no 3′UTR binding was detected by luciferase reporter assays. In contrast, a 7mer-A1 seed sequence match for the CDS region (see Table 1) was detected. In total, 15 out of the targeted proteins have at least one putative miR-155 binding site, as identified by bioinformatic analysis.

Next, we analysed the binding of miR-155 to the mRNA sequences of our identified target set. Luciferase reporter assays using the 3′UTR sequences of KIF11, CKAP5 and IKBKE were performed. IKBKE served as a positive control (see Figure 4), since miR-155 3′UTR sequence binding was previously reported [39]. For KIF11 and CKAP5, regulation was detected at the protein level by SILAC, as well as by Western blot analysis, but only the 3′UTR region of CKAP5 is targeted by miR-155 using luciferase sensor constructs (see Figure 4). Table 1 summarized the data for the protein level, mRNA level and target sequence binding for all significant, differentially expressed proteins analysed.

### Functional annotation to elucidate the role of miR-155 target proteins

To gain further insight into the possible biological role of the 46 target proteins, regulated by miR-155, a bioinformatic analysis using the DAVID2008 functional analysis software package was performed [37], [38]. We used the functional annotation clustering approach, which reduces the redundancy in annotation by grouping similar annotations consisting of similar sets of proteins.

In Table 2, the most significant functional gene ontology (GO) annotation cluster (cluster 1-cell cycle processes) is shown with its respective proteins. Twelve of the 46 miR-155 target proteins are represented in this cluster, including the proteins CKAP5, KIF11, UBE2C and KPNA2, which were identified as miR-155 target proteins by SILAC and Western blot analysis in the present study. CKAP5 has been identified as a direct target of miR-155 and PCM1, KIF11, RBM4 and TUBA1C are putative direct targets of miR-155, due to their detection by prediction algorithms or seed sequence matches. Further clusters include, among others, stimulus response and RNA processing. The complete DAVID analysis is found in Supplementary Table S5.

**Table 2:** Functional annotation of potential miR-155 target proteins in HEK293T cells.

	CKAP5	KIF11	UBE2C	CENPE	KPNA2	PCM1	TUBA1C	RBM4	TIMM17A	RPS14	GPS1	MAPK14
Microtubule-based process	X	X	X	X	X	X	X					
Centrosome organization	X	X				X						
Microtubule organizing center organization	X	X				X						
Microtubule cytoskeleton organization	X	X	X			X						
M phase	X	X	X	X	X							
Nuclear division	X	X	X	X								
Mitosis	X	X	X	X								
M phase of mitotic cell cycle	X	X	X	X								
Organelle fission	X	X	X	X								
Cell division	X	X	X	X								
Cytoskeleton organization	X	X	X			X						
Cell cycle	X	X	X	X	X						X	X
Mitotic cell cycle	X	X	X	X	X							
Cell cycle phase	X	X	X	X	X							
Cell cycle process	X	X	X	X	X							
Organelle organization	X	X	X	X		X		X	X			
Cellular component organization	X	X	X	X		X	X	X	X	X		

## Discussion

MicroRNA research is currently focusing on the identification of new miRNAs [40], the elucidation of their relative expression levels [41], their functional effects in diseases [42] and their therapeutic implications [43]. The missing link is often the identification of microRNA targets and their biological functions. So far, miRNA target identification is restricted, because microRNAs can exert their effects at either the protein or mRNA level, and the degree to which each can occur in a specific tissue is still a matter of debate. In addition, large scale quantitative proteomic studies have only been conducted using a limited number of miRNAs and cell lines [5]–[7], [15], [16], [18].

This study was performed to identify miR-155 target proteins in HEK293T cells using quantitative proteomics and to connect these data to functional annotations. A 48 hour time period after transfection was used for the analysis due to the strong miR-155 expression found during this time. Forty-six potential miR-155 target proteins were identified in HEK293T cells using the SILAC approach upon miR-155 overexpression, and five selected targets were further confirmed by quantitative Western blot analysis. The changes of protein expression were subtle, which is in agreement with the hypothesis that single miRNAs act as rheostats by fine tuning protein abundance [18]. Although all five of the proteins analyzed by Western blot were downregulated to a certain degree, only KIF11 and CKAP5 passed statistical analysis (Figure 3). This might mean that only strongly deregulated proteins show statistical relevance under the conditions used in these experiments. We also compared our proteomic data with the mRNA expression values of the selected targets (see Figure 3). These results support the view that the translational inhibition of miRNAs might be a major mechanism of protein downregulation [5], [6], [7]. However, our choice of a 48 hour time period for measurement may have increased the number of proteins indirectly modulated by miR-155, resulting in the production of more proteins not containing seed sequences.

The view that microRNAs might exert their effects mainly by translational inhibition is quite controversial. Several authors have shown an inverse correlation between microRNA modulation and mRNA expression in genes containing putative microRNA target sequences [12]–[14]. More recently, Guo et al. [15] performed large scale sequence analysis of both total mRNA and ribosome bound mRNA transcripts in, among others, miR-155 overexpressing HeLa cells, correlated these results to protein data determined by SILAC analysis from a previous miR-155 overexpression study [5] and confirmed that an inverse correlation existed between microRNA modulation and gene expression. In addition, in this study, comparison of genes containing miR-155 targets determined by bioinformatic analysis showed little decrease in ribosomally bound genes when compared to total mRNA, suggesting that translational inhibition was not the main mechanism in the regulation of these genes.

In contrast, several other studies have shown that specific microRNAs inhibit either the initiation or elongation step of protein translation [8], [44], [45]. On a larger scale, analysis of miR-21 overexpressing MCF7 breast cancer cells by expression microarray and protein analysis using an iTraq based proteomics strategy showed no strong correlation between mRNA and protein expression [6]. Recently, a similar study of miR-143 overexpression in a pancreatic carcinoma cell line from the same laboratory using microarray- and SILAC analyses gave similar results [16].

The interaction of miRNAs with their target mRNA sequences is not completely understood. Target prediction algorithms try to look at different kinds of binding characteristics (seed sequences matches, thermodynamics, sequence conservation or a combination of these) [17]. It was shown that, besides the most frequent 3′UTR binding, also the 5′UTR binding [46], [47] or binding to the CDS region [47] must be considered.

We, therefore, examined potential miRNA targets identified by large scale proteomics for their miRNA binding in the CDS and 5′UTR by using the RNA22 algorithm [32] and seed sequence matches in these regions (see Table 1 and 3). For several target proteins, the RNA22 algorithm or seed sequence matches indicated a binding of miR-155 in the CDS, which is in agreement with the high percentage of miRNA binding sites in CDS in a recently published Argonaut Co-IP mRNA-miRNA interaction screen [48].

**Table 3:** Potential upregulated miR-155 target proteins in HEK293T cells detected by SILAC with prediction algorithms and seed sequence matches.

HGNC symbol	SILAC	Prediction algorithms and seed sequence matches	HGNC symbol	SILAC	Prediction algorithms and seed sequence matches
SMC1A	1,133	CDS 7mer-A1	LMNA	1,227	n.d.
C8orf62	1,141	n.d.	SUCLG2	1,234	3′ UTR 7mer-A1
HIST1H1C	1,146	n.d.	PSAP	1,235	n.d.
LMNB1	1,149	n.d.	CYP51A1	1,256	n.d.
GARS	1,154	n.d.	SAMM50	1,27	n.d.
HDGF	1,158	n.d.	CRABP2	1,277	n.d.
CDC37	1,165	3′UTR-7mer-m8	PAPSS1	1,278	n.d.
CA2	1,167	n.d.	TMPO	1,302	CDS 7mer-A1
PWP2	1,176	n.d.	PSIP1	1,308	n.d.
PYCR1	1,178	n.d.	AMOT	1,328	n.d.
LMNB2	1,189	n.d.	CHCHD2	1,365	n.d.
DARS2	1,191	n.d.	DPYSL2	1,369	n.d.
ACSL3	1,198	CDS 7mer-A1, 3′UTR 7mer-m8	TRIO	1,371	CDS 8mer
CLIC4	1,203	CDS 7mer-A1	CTH	1,436	n.d.
RFC3	1,205	n.d.	DHFR	1,437	CDS 7mer-A1
DDX52	1,206	CDS 7mer-m8	H1FX	1,439	n.d.
ALDH7A1	1,207	n.d.	XPNPEP1	1,528	CDS 8mer
DYNC1I2	1,216	n.d.	EMD	1,62	n.d.
NDUFS1	1,218	n.d.	CABC1	1,654	n.d.
TYMS	1,221	n.d.	PCK2	1,701	n.d.
TFB1M	1,222	n.d.	PDCD4	1,71	3′UTR 8mer

_a_HGNC gene symbols (www.genenames.org);

_b_fold change miR-155/pCMX;

_c_Analyzed using RNA22 CDS, 3′UTR, 5′UTR; DIANA microT, TargetScan, MicroCosm;

_d_Seed sequence (8mer, 7mer-A1, 7mer-m8) matches in the different mRNA regions (5′UTR, CDS, 3′UTR);

_e_RN22-CDS positive;

_f_RNA22-3′-UTR positive; -: not tested; n.d.: not detected; *:

We found that overexpression of miR-155 leads predominantly to changes in proteins associated with the cell cycle (Table 2), which was also shown previously by the miR-155 target protein TP53INP1 [49] and could now be extended and linked to new cell cycle mediators. For example, CKAP5, verified as a primary miR-155 target protein by luciferase reporter assay in this study, plays a role in spindle pole integrity and is controlled by Aurora-A which is one of the major checkpoints of the cell cycle [50]. The decrease of KIF11 can inhibit proliferation, increase apoptosis rate and alter the expression of cell cycle regulating proteins like survivin and Aurora-A [51]. It has also been shown that KIF11 inhibits the destruction of cyclin A and B, arrests cells in M phase and impedes the onset of anaphase by blocking the ubiquitin-dependent proteolysis of proteins responsible for sister chromatid separation [52].

So far, 26 direct miR-155 targets were experimentally validated [21]. We quantified two of them, namely RHOA and FADD, but both showed no significant downregulation in HEK293T cells. RHOA has been validated in NMuMG cells [53] and FADD in MCF7 cells [39]. This indicates that miRNA targets seem to be cell line specific, as previously reported for single proteins [20]. Grosshans and Filipowicz suggested a re-analysis of the existing proteomic data of miRNA target identification with respect to method reliability by utilization of different approaches [54]. To compare our data set of miR-155 target proteins in HEK293T cells with others, we chose the only available large scale quantitative proteomic data for miR-155 targets, which were generated in HeLa cells [5]. About 1/3 of the proteins downregulated in HeLa were also quantified in HEK293T cells (35 of 100). However, none of these proteins were detected as a miR-155 target in HEK293T cells. One possible explanation might be that our experimental approach differed substantially from the one applied by Selbach et al, 2008. First, we used a vector system for miRNA overexpression, whereas Selbach et al used synthetic miRNAs. Synthetic miRNAs have an enhanced stability and circumvent, in contrast to precursor miRNAs, parts of the natural biogenesis [55]. It is not known, whether this might influence miRNA target proteins. Second, the SILAC workflow itself was different: Selbach et al, 2008 used the so-called pSILAC approach for pulsed labelling of *de-novo* synthesized proteins, whereas we performed the classical SILAC workflow. To investigate the influence of different workflows, we compared the pSILAC [5] with the classical approach [7], [18]. All three studies aimed to detect miR-1 targets in HeLa cells by synthetic miRNAs. The overlap of downregulated proteins is 50% between Vinther et al, 2006 and Selbach et al, 2008; 25% between Vinther et al, 2006 and Baek et al, 2008 and 18% between Selbach et al, 2008 and Baek et al, 2008. Thus, the overlap of identified microRNA targets using different SILAC approaches was substantial. Excluding the workflow dependency, we demonstrated by comparison of miR-155 target proteins in HEK293T and HeLa cells the cell line specificity of the respective microRNA targets. One possible explanation for the specificity of miRNAs could be the differential expression of mRNAs and their function as pseudotargets [56].

In summary, we identified novel target proteins of miR-155 in HEK293T cells and showed that these proteins are predominantly involved in cell cycle regulation. In addition, this study indicates that microRNA target proteins seem to be cell line specific to a larger extent than previously known.

## Supporting Information

Figure S1 **Relative expression of miR-155.** Expression of miR-155 was measured 48 h after transfection of HEK293T cells with miR-155 pCMX vector or the empty pCMX vector. Cells transfected with miR-155 showed a ca. 1000 fold overexpression compared to the empty vector control.
(TIF)

Figure S2 **Western blot image of CKAP5.** HEK293T cells were transiently transfected with miR-155 or empty vector (pCMX). Four independent biological replicates were analysed. Secondary antibodies were labelled with CyDyes. Intensities of GAPDH were used for normalization.
(TIF)

Table S1 **List of oligonucleotides for miR-155 overexpression construct and for sensor constructs, oligonucleotide sequences for qRT-PCR assay and list of antibodies used for Western blotting.**
(XLS)

Table S2 **List of downregulated proteins identified and quantified by SILAC.**
(XLS)

Table S3 **List of upregulated proteins identified and quantified by SILAC.**
(DOC)

Table S4 **Detailed information on the five proteins KIF11, UBE2C, RANGAP1, KPNA2 and CKAP5 shown in** **Figure 3** **.** Data on identified and quantified peptides are given as obtained by MaxQuant 1.0.13.13. Only peptides unique to the respective protein are listed.
(XLS)

Table S5 **Functional annotation of potential miR-155 target proteins using the DAVID2008 analysis software.**
(XLS)
