# Supplementary figures and images for: Quantitative Proteomics Identify Novel miR-155 Target Proteins
Source: PLoS One. 2011 Jul 25;6(7):e22146. doi: 10.1371/journal.pone.0022146 (PMC3143118; doi:10.1371/journal.pone.0022146)

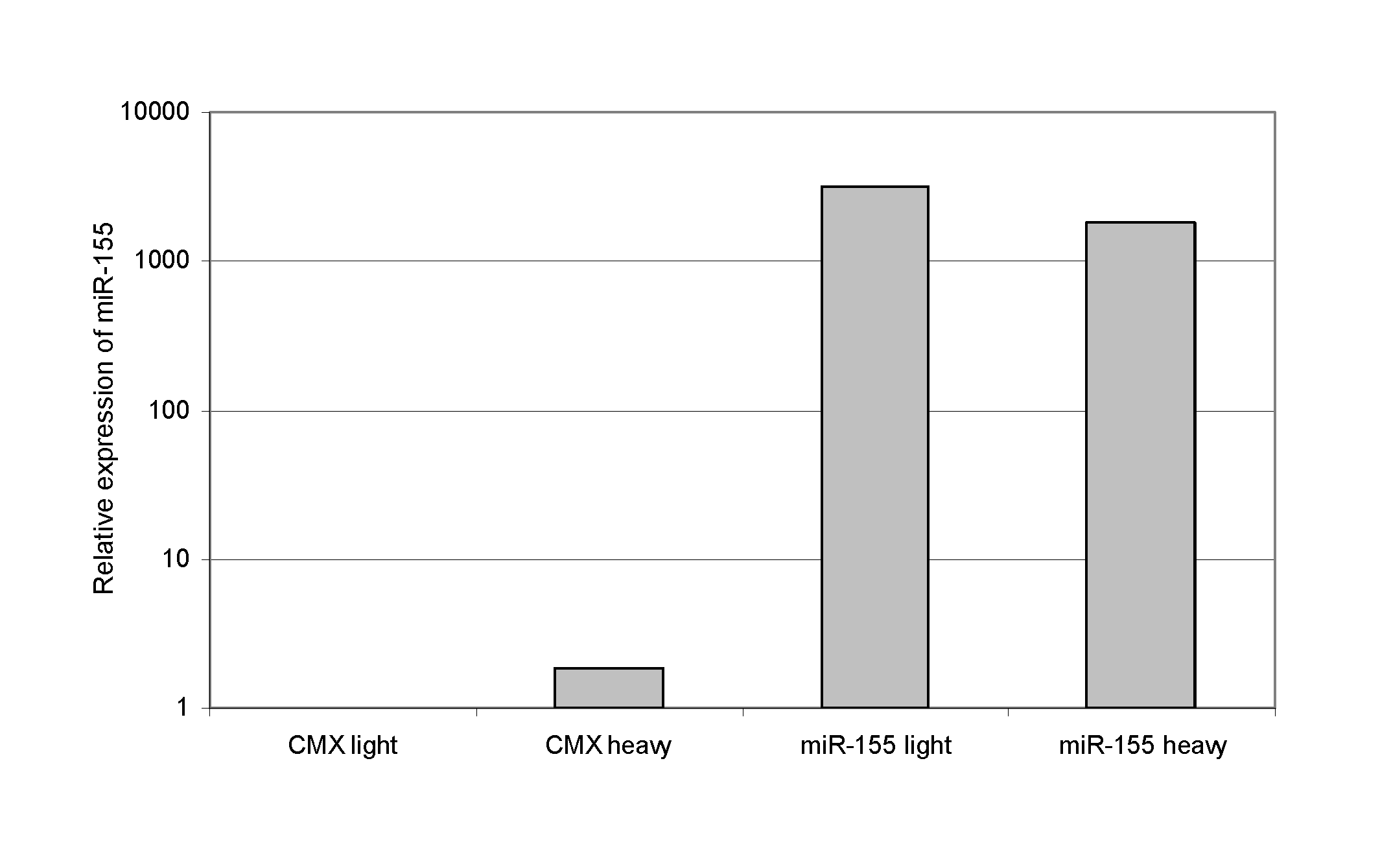

Supplement: Figure S1 — Relative expression of miR-155. Expression of miR-155 was measured 48 h after transfection of HEK293T cells with miR-155 pCMX vector or the empty pCMX vector. Cells transfected with miR-155 showed a ca. 1000 fold overexpression compared to the empty vector control. (TIF) [file pone.0022146.s001.tif]

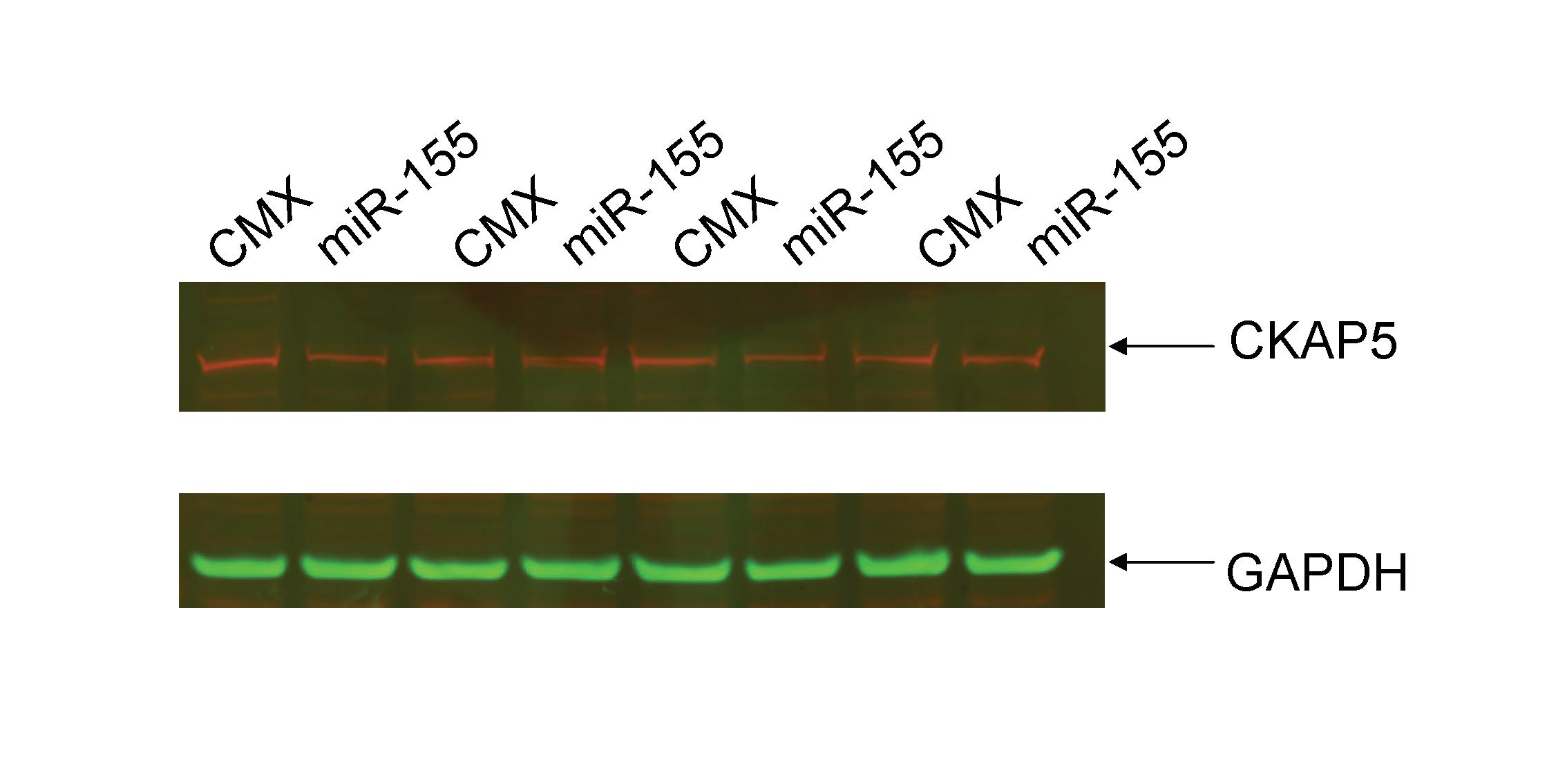

Supplement: Figure S2 — Western blot image of CKAP5. HEK293T cells were transiently transfected with miR-155 or empty vector (pCMX). Four independent biological replicates were analysed. Secondary antibodies were labelled with CyDyes. Intensities of GAPDH were used for normalization. (TIF) [file pone.0022146.s002.tif]
