# Supplementary material for: Quantitative Proteomics Identify Novel miR-155 Target Proteins
Source: PLoS One. 2011 Jul 25;6(7):e22146. doi: 10.1371/journal.pone.0022146 (PMC3143118; doi:10.1371/journal.pone.0022146)
Supplement: Table S3 — List of upregulated proteins identified and quantified by SILAC. (DOC) [file pone.0022146.s005.doc]

| **Oligonucleotides for miR-155 overexpression construct** | | | | |
| --- | --- | --- | --- | --- |
| **microRNA** | **Reference sequence**  **number** | **Oligonucleotide sequences** | **Size of product**  **(bp)** | **Optimal**  **annealing**  **temperature (°C)** |
| miR-155 | nr-001458  ([chr21:26946247+26946411](http://genome.cse.ucsc.edu/cgi-bin/hgTracks?hgsid=148093284&db=hg19&position=chr21:26946247-26946411&hgPcrResult=pack)) | CAGAATTCATCCTCTGAGTGCTGAAGGCT  CACTCGAGACGGCAGCAATTTGTTCCATGT. | 165 | 70 |
| **Oligonucleotides for sensor constructs** | | | | |
| **Gene name** | **Reference sequence**  **number** | **Oligonucleotide sequences** | **Size of product**  **(bp)** | **Optimal**  **annealing**  **temperature (°C)** |
| KIF11 (3’ UTR) | nm_004523  ([chr10:94413561+94415150](http://genome.cse.ucsc.edu/cgi-bin/hgTracks?hgsid=148093284&db=hg19&position=chr10:94413561-94415150&hgPcrResult=pack)) | TTTACTAGTGGGGGTTGGCAATTTTATTT  TTTGAGCTCAATGTAGAAACCACATTTATTAACCA | 1590 | 60 |
| CKAP5 (3’ UTR) | nm_01456  nm_001008938 | TTTACTAGTAGAGCAGTCGCAAATGAAGC  TTTAAGCTTATGGAGCATCTTGGGAGTCA | 522 | 61 |
| IKBKE (3’ UTR) | nm_014002 | TTTACTAGTGCACATGAGGCATCCTGAA  TTTGAGCTCAAGTCCTATAAACCAGAGGGAGTG | 351 | 60 |
| **Oligonucleotide sequences for qRT-PCR assay** | | | | |
| **Gene name** | **Reference sequence**  **number** | **Oligonucleotide sequences** | **Size of product (bp)** | **Optimal**  **annealing**  **temperature (°C)** |
| UBE2C | nm_007019 | ggtgggcaaaaggctaca  aatccctttatcgccagaca | 61 | 56 |
| KPNA2 | nm_002266 | ctgcaggaaaaccgcaac  tgctgctatttatgcctttgac | 76 | 55 |
| KIF11 | nm_004523 | catccaggtggtggtgagat  tattgaatgggcgctagctt | 67 | 56 |
| CKAP5 | nm_001008938 | aaaaggcacctgctgctaag  tcctggtgcagctggttt | 62 | 59 |
| RANGAP1 | nm_002883 | cggaagattctggaccctaa  aggaggtggggaggacag | 60 | 60 |

| **Antibodies for Western blotting** | | | | |
| --- | --- | --- | --- | --- |
| **Name (HGNC symbol)** | **Product number** | **Supplier** | **Host** | **Dilution** |
| KIF11 | A301-075A | Bethyl Laboratories (Montgomery, USA | Rabbit | 1:2000 |
| UBE2C | ab12290 | Abcam (Cambridge, UK) | Rabbit | 1:500 |
| RANGAP1 | 11102-1-AP | Proteintech Group (Chicago, USA | Rabbit | 1:500 |
| KPNA2 | ab54489 | Abcam (Cambridge, UK) | Rabbit | 1:1000 |
| CKAP5 | PA3-16835 | Thermo Fisher Scientific (Waltham, USA) | Rabbit | 1:1000 |
| GAPDH | CB1001 | EMD Chemicals (Gibbstown, USA) | Mouse | 1:10000 |
| ECL Plex goat-α-mouse IgG-Cy3 | PA43009 | GE Healthcare Bio-Sciences, Uppsala, Sweden | Goat | 1:2500 |
| ECL Plex goat-α-rabbit IgG-Cy5 | PA45011 | GE Healthcare Bio-Sciences, Uppsala, Sweden | Goat | 1:2500 |
